# Noise‐Aware Active Learning to Develop High‐Temperature Shape Memory Alloys with Large Latent Heat

**DOI:** 10.1002/advs.202406216

**Published:** 2024-10-03

**Authors:** Yuan Tian, Bin Hu, Pengfei Dang, Jianbo Pang, Yumei Zhou, Dezhen Xue

**Affiliations:** ^1^ Materials Genome Institute Shanghai University Shanghai 200444 China; ^2^ School of Materials Science and Engineering Shanghai Jiao Tong University Shanghai 200240 China; ^3^ State Key Laboratory for Mechanical Behavior of Materials Xi'an Jiaotong University Xi'an 710049 China

**Keywords:** active learning, latent heat, noise level estimation, phase change materials, thermal energy storage

## Abstract

Shape memory alloys (SMAs) with large latent heat absorbed/released during phase transformation at elevated temperatures benefit their potential application on thermal energy storage (TES) in high temperature environment like power plants, etc. The desired alloys can be designed quickly by searching the vast component space of doped NiTi‐based SMAs via data‐driven method, while be challenging with the noisy experimental data. A noise‐aware active learning strategy is proposed to accelerate the design of SMAs with large latent heat at elevated phase transformation temperatures based on noisy data. The optimal noise level is estimated by minimizing the model error with incorporation of a range of noise levels as noise hyper‐parameters into the noise‐aware Kriging model. The employment of this strategy leads to the discovery of the alloy with latent heat of –36.08 J g^−1^, 9.2% larger than the best value (–33.04 J g^−1^) in the original training dataset within another four experiments. Additionally, the alloy represents high austenite finish temperature (481.71°C) and relatively small hysteresis. This promotes the latent heat TES application of SMAs in high temperature circumstance. It is expected that the noise‐aware approach can be convenient for the accelerated materials design via the data‐driven method with noisy data.

## Graphical abstract

A noise‐aware active learning strategy is introduced for the data‐driven materials design under the potential impact of data noise. The optimal noise parameter is estimated by minimizing the model errors. Integrating the optimal noise parameter into modeling and query steps leads to a high efficiency in discovering materials with the desired properties.

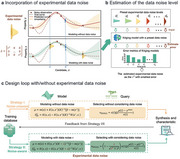

## 1 Introduction

Shape memory alloys (SMAs), a subset of metallic phase change materials, possess the capability to store and release thermal energy through solid‐solid martensitic phase transformation.^[^ ^1^ ^]^ This characteristic renders them promising for applications in latent heat thermal energy storage (LHTES).^[^ ^2^, ^3^ ^]^ NiTi‐based alloys, with their high thermal conductivity, exceptional corrosion resistance, minimal volume change, and substantial volumetric heat storage capacity, stand out among various shape memory alloys, making them ideal candidates for LHTES.^[^ ^4^ ^]^ However, diverse applications may require energy storage solutions tailored to specific temperatures.^[^ ^5^ ^]^ For example, high‐temperature applications, as found in solar power plants, steam generators and industrial waste‐heat recovery, demand a substantial latent heat during martensitic transformation at elevated temperatures, which well surpass the phase transformation temperatures of 100°C limitation for NiTi binary alloys.^[^ ^4^, ^6^ ^]^ Hence, there is a need to identify NiTi‐based compositions with high latent heat at elevated phase transformation temperatures.

Modifying the Ni and Ti balance and/or introducing doped elements allows optimizing latent heat and tuning the transformation temperature within a wide range from –50 to 500°C.^[^ ^7^ ^]^ As the complexity and size of alloy elements increase, a multitude of potential alloys emerges, leading to difficulties in efficiently exploring this expansive composition space while minimizing experimental costs.^[^ ^8^ ^]^ The data‐driven approach, learning from previous data to predict materials properties, offers a potential solution for the rapid design of new materials with desired performance.^[^ ^9^, ^10^, ^11^, ^12^, ^13^, ^14^, ^15^ ^]^ However, the majority of data collected for alloys from laboratory experiments or literature typically consists of only a single‐time measured value, and often regarded as a noisy observation of the true value.^[^ ^16^, ^17^ ^]^ These noisy data points inevitably serve as inputs for machine learning, potentially leading to a degradation in machine learning performance and increased uncertainties associated with predictions.^[^ ^18^, ^19^ ^]^ Therefore, it is crucial to reduce the impact of such noise‐contained experimental data.

Recent efforts have been devoted to reducing the influence of noise in experimental data through pre‐cleaning techniques or utilizing machine learning models with high noise tolerance.^[^ ^20^, ^21^ ^]^ Noise filters designed to identify and rectify outliers before training the predictive model have been employed.^[^ ^22^ ^]^ For instance, studies by Khoshgoftaar et al. and Liu et al. have demonstrated that predictive model performances often improve when trained on datasets that have undergone filtering.^[^ ^23^, ^24^ ^]^ However, it is challenging to entirely eliminate experimental noise using data cleaning methods alone. Robust algorithms less sensitive to data noise are further employed to predict materials properties.^[^ ^25^ ^]^ For example, Reinbold et al. have shown that symbolic regression, coupled with domain knowledge, enables the creation of accurate models even with high‐dimensional, noisy, and incomplete data.^[^ ^26^ ^]^ Zhang et al. utilizes ensemble learning algorithms that can bear the data noise to achieve better predictions of HEA hardness.^[^ ^27^ ^]^ Nevertheless, in scenarios of elevated data noise, even robust models may exhibit suboptimal performance.

Active learning framework or Bayesian optimization provide suitable means of regarding data noise level as a parameter to be integrated into modeling and query steps, which can accelerate the materials design via making use of the prediction uncertainty arising from the sparse distribution of the training data as well as data noise.^[^ ^28^, ^29^ ^]^ The prediction uncertainties due to sparse data distribution enrolled in active learning loop have been used successfully in accelerating the discovery of various materials, including epitaxial nanocomposite phase‐change memory material,^[^ ^30^ ^]^ shape memory alloys with small hysteresis,^[^ ^8^ ^]^ and high‐entropy Invar alloys with low thermal expansion coefficients.^[^ ^31^ ^]^ However, data noise has seldom been taken into account as a factor contributing to the uncertainties associated with machine learning predictions in these studies.^[^ ^32^, ^33^ ^]^

The evaluation of data noise level (i.e., the data uncertainty) typically relies on two types of approaches. It can be achieved through scientific judgement based on a pool of comparatively reliable information, including uncertainties assigned to reference data from handbooks, calibration and other certificates, experience or general knowledge of relevant materials and instruments, etc.^[^ ^34^ ^]^ Nevertheless, the absence of universally accessible expert handbooks or comprehensive experience for numerous materials properties often hinders such kind of noise level estimation. Alternatively, the noise level can be estimated through the statistical analysis of repeated measurements conducted on several parallel samples.^[^ ^21^, ^35^ ^]^ However, determining the uncertainties for a comprehensive SMAs database from parallel testing results poses challenges, given the time‐consuming and expensive nature of most experimental measurements. Therefore, a critical challenge is how to evaluate the optimal data noise level parameters in the active learning without involving additional experiments, and make use of experimental noise to enhance the efficiency of discovering new materials.

In the present study, we present an experimental data noise‐aware active learning approach for developing SMAs with large latent heat at elevated temperatures even based on noisy experimental data. The strategy comprises two main components. Part I estimates the noise level by minimizing the noise‐aware Kriging model error across a range of noise levels. Subsequently, Part II involves training a Kriging model with the noise level as an input to establish correlations between latent heat and materials descriptors. Additionally, a noise‐augmented acquisition function as the query strategy is employed when determining the next alloy composition. This strategy led to the discovery of a Ti_0.266_Ni_0.484_Hf_0.25_ alloy with latent heat of –36.08 J g^−1^ within four experimental iterations out of a search space of 10^6^. The alloy exhibits a high austenite finish temperature (481.71 °C) and relatively small hysteresis, beneficial for LHTES applications in high‐temperature circumstances. More importantly, the efficiency of the noise‐aware design loop is proved much higher than that without considering experimental noise through comparison. These results suggest that quantifying the noise level of data and utilizing it can aid in the data‐driven development of new materials.

## 2 Results and Discussion

### 2.1 Design Strategy

#### 2.1.1 Experiment Data Noise‐Aware Active Learning Strategy

Usually, noisy observations $\tilde{y_{i}}$ of a materials property are probabilistically distributed around the true value *y*_*i*_ within a specified range. It can be denoted as $\tilde{y_{i}}=y_{i}+\varepsilon_{i}$, in which $\varepsilon_{i}$ represents a single realization of a noise random variable ϵ_*i*_. The noise variable ϵ_*i*_ is assumed to follow a Gaussian distribution, $N(0,\tau^{2})$, where τ^2^ is the variance of noise. The range of the noise level could vary when deal with different kinds of materials property measurement problems, such as a maximum hardness noise standard deviation value of 67 HV in Wen's work^[^ ^35^ ^]^ and a maximum yield strength noise standard deviation value of 153.58 MPa in Zhang's work.^[^ ^32^ ^]^ In this study, the actual data noise is unknown. To better match the range of noise level to the specific materials problem, we calculate the difference between the maximum and the minimum property values (*y* ^*max*^ − *y* ^*min*^) in the existing data, and define the noise variance as τ^2^ = [ϕ(*y* ^*max*^ − *y* ^*min*^)]^2^, where ϕ ∈ (0, 1) is a prefactor to control the magnitude of noise level. The noise level set in this way can be incorporated into the modeling and optimal design strategy in active learning framework, interfering with the impact of data noises on model predictions and the querying results.

**Figure 1:**
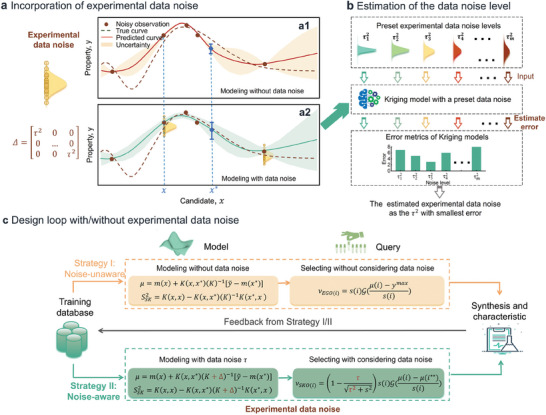
Schematical figure of experiment data noise‐aware active learning strategy. a) Predicted results and the associated uncertainties in the cases of data noise‐unaware and data noise‐aware. The normal distribution represents the distribution of random property observations of one candidate. b) The flow chart of experiment noise level estimation. c) Noise‐aware active learning loop compared with noise‐unaware loop.

*Noise‐aware Kriging model*. A Kriging model that takes the data noise into consideration can be employed to provide a better prediction of the latent heat property value. In the noise free case, the covariance *K* between training data points can be obtained using a kernel function, such as the Gaussian kernel $g\left(h\right)=\exp\left(-\frac{1}{2}{\left(\frac{h}{\theta }\right)}^{2}\right)$, where *h* and θ are the hyperparameters. In the presence of noise, the covariance is modified to *K* + **Δ**, where **Δ** is a diagonal matrix with diagonal terms representing the variance τ^2^ associated with measurement noise, as shown in **Figure** **1**a. The predicted mean value (μ) and prediction uncertainties (*s* ^2^) can then be expressed as follows:^[^ ^36^, ^37^ ^]^

(1) $$\mu =f{\left(x\right)}^{⊺}\xi +K{\left(x^{*},x\right)}^{⊺}{\left(K+\Delta \right)}^{-1}\left(\tilde{y}-f\left(x^{*}\right)\xi \right),$$

(2)

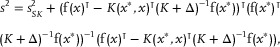

where noisy observations $\tilde{y}={\left({\tilde{y}}_{1},\text{…},{\tilde{y}}_{p}\right)}^{T}$, *p* is the total count of labeled training data points $x^{*}$, x represents the unknown data points, f(*x*) is the vector of trend function values, f(*x* ^*^) is experimental matrix, ξ is the vector of coefficients. And $s_{SK}^{2}$ is the variance given by Simple Kriging (SK), the formula is shown by:

(3) $$s_{\mathrm{SK}}^{2}=K\left(x,x\right)-K{\left(x^{*},x\right)}^{⊺}{\left(K+\Delta \right)}^{-1}K\left(x^{*},x\right)$$

Figure 1a schematically illustrates the comparison between data noise‐unaware and data noise‐aware situations. The introduction of experimental noise alters the predicted property values of samples, as seen by the change in the solid line from Figure 1(a1) to Figure 1(a2). Importantly, the prediction uncertainty given by noise‐aware model arises from both sparse data and data noise, while the prediction uncertainty given by noise‐unaware model arises only from sparse data. Thus, the estimated uncertainties associated with these predictions also vary, as indicated by the shaded areas in Figure 1(a1) to Figure 1(a2). Consequently, besides the predicted values, the selection of the next experiment, whether based on prediction, uncertainty, or a trade‐off between the two, will be affected.

*Estimation of experimental noise level*. As the experimental noise is unknown, we first estimate the experimental noise level without using the high costs and time‐consuming repeated‐testing. The estimation method upon the existing database is schematically shown in Figure 1b. We set a series of noise levels $T=\{\tau_{1}^{2},\tau_{2}^{2},\text{…},\tau_{m}^{2}\}$ covering a wide range. Each component of T is input into the noise‐aware Kriging model as noise matrix (**Δ**) and the error of the Kriging model is estimated. The τ^2^ value giving the minimal error is considered as the optimal noise level.

Two error metrics of the Kriging model are calculated, allowing us to track the evolution of the model performance with the input of noise level $\tau_{m}^{2}$. The first metric is the Mean Squared Error (MSE), calculated by $\mathrm{MSE}=\frac{\sum_{i=1}^{n}{\left(\tilde{y_{i}}-\mu_{i}\right)}^{2}}{n}$, where $\tilde{y_{i}}$ is the observed value and µ_*i*_ is the estimated mean value from the Kriging model. As $\tilde{y_{i}}$ is considered as a noisy observation of the true value, MSE may not truly describe the model accuracy. Hence, the Continuous Ranked Probability Score (CRPS) is employed as the second metric for its uncertainty‐aware nature. The CRPS is defined as,

(4) $$\begin{array}{c} CRPS=s\left(\frac{1}{\sqrt{\pi }}-2\psi \left(\frac{\tilde{y_{i}}-\mu }{s}\right)-\frac{\tilde{y_{i}}-\mu }{s}\left(2\Psi \left(\frac{\tilde{y_{i}}-\mu }{s}\right)-1\right)\right) \end{array}$$

where ψ and Ψ are the probability density function and the associated cumulative distribution function.

*Active learning considering noise*. As depicted Figure 1c, Strategy I is noise‐unaware and represents a sequential optimization loop that disregards the impact of experimental noise. It utilizes a Kriging model without incorporating experimental noise to estimate the mean value (μ) and associated uncertainties (*s* ^2^) of candidates in the search space. Based on these estimates, an acquisition function of Efficient Global Optimization (EGO) is employed to score the unknown alloys, guiding the selection of potential candidates for next experiments. EGO maximizes the expected improvement over the best observed value $y^{max}$ so far:^[^ ^38^, ^39^ ^]^

(5) $$\nu_{EGO}=sG\left(\frac{\mu -y^{max}}{s}\right)$$

where G is a mathematical operator given by *z*Ψ(*z*) + ψ(*z*), ψ is the probability density function of a standard normal distribution, Ψ is the associated cumulative distribution function and $z=\frac{\mu -y^{max}}{s}$. Note that the EGO does not contain information on the experimental noise level τ^2^. Consequently, noise‐unaware Strategy I disregards experimental noise considerations in both the modeling and querying steps.

In contrast, noise‐aware Strategy II in Figure 1c employs the Kriging model as per Equation (2), which incorporates the estimated experimental noise level τ^2^. Meanwhile, a noise‐augmented acquisition function of Sequential Kriging Optimization (SKO) is utilized to suggest the next experiment. This function is expressed as:^[^ ^40^ ^]^

(6) $$\nu_{\mathrm{SKO}}=\left(1-\frac{\tau }{\sqrt{\tau^{2}+s^{2}}}\right)sG\left(\frac{\mu -\mu^{**}}{s}\right)$$

where μ^**^ = μ_(argmax[μ − λs])_, λ is the “risk‐avoid” parameter, $1-\frac{\tau }{\sqrt{\tau^{2}+s^{2}}}$ is a prefactor introduced to consider the experimental noise. The comparison between Strategy I and Strategy II would reveals the role of experimental noise in optimization of materials properties.

#### 2.1.2 Assessing the Feasibility and Robustness of the Noise‐Aware Strategy

**Figure 2:**
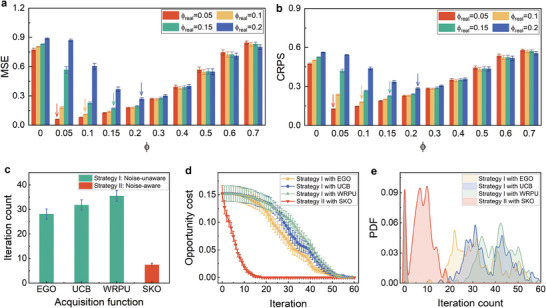
a) MSE and b) CRPS as a function of the input noise level parameter prefactor ϕ over 200 trials. Different colors represent different prefactors ϕ_*real*_ of the true noise levels. The optimal noise parameter prefactors that yield the minimum MSE and CRPS are pointed out by arrows. c) The iteration count to identify the option with the lowest $\tilde{y}$ value varies with the method employed. The cyan bars depict the outcomes of Strategy I that does not consider noise in the model or acquisition functions, the red bar represents the result achieved by Strategy II (noise‐aware) introduced in this work. d) The opportunity cost changes as the iterations increase. A significant trend of declining opportunity cost implies a faster approach toward the goal. e) Probability density functions (PDF) are represented as functions of the iteration count. When the peak of the distribution is closer to an iteration count of 0, it indicates that the strategy demonstrates a higher efficiency in the majority of the 200 trials.

The feasibility and robustness tests of the noise‐aware strategy are conducted on a well‐known 3D Hartmann3 function characterized by the presence of multiple local minima and a global minimum. The formula for Hartmann3 function is shown in Equation S2 (Supporting Information). We first construct the database with noisy observations using real noise. The whole space is discretized into 400 points using Latin Hypercube Sampling, and the corresponding true values *y*_*i*_ are calculated by the Hartmann3 function. The noisy observation ${\tilde{y}}_{i}$ is sampled from a Gaussian distribution $N(y_{i},\tau_{i}^{2})$, τ_*i*_ = ϕ_*real*_ · (*y* ^*max*^ − *y* ^*min*^), as detailed in S1 (Supporting Information). ϕ_*real*_ denote as the true noise level prefactors, and are assigned values of 0.05, 0.1, 0.15, and 0.2. From the entire dataset, 15% data are randomly chosen to train a Kriging model with noise level [ϕ · (*y* ^*max*^ − *y* ^*min*^)]^2^, where the prefactor is preset as ϕ = 0, 0.05, 0.1, 0.15, 0.2, 0.3, 0.4, 0.5, 0.6, 0.7. Subsequently, the MSE and CRPS are calculated using all 400 data points available and ϕ corresponding to the minimum MSE/CRPS is decided as the optimal prefactor ϕ_*opt*_. The statistical results of MSE and CRPS over 200 trails are represented in the bar plots of **Figure** **2**a,b. When the true noise level ϕ_*real*_ is set to 0.05, the optimal prefactor ϕ_*opt*_ is estimated as 0.05 in agreement with ϕ_*real*_. Similarly, when ϕ_*real*_ is set to other values, the estimated ϕ_*opt*_ values remain consistent with those ϕ_*real*_. ϕ_*opt*_ → ϕ_*real*_ indicates a proper noise level estimation, which proves that it is able to approximate the real noise level by evaluating the optimal prefactor ϕ_*opt*_. To gain a clear visual understanding of how the noise level parameter affects prediction results, an investigation is undertaken on a 1D mathematical function in Equation (S1), Figures S1– S4, and Table S1 (Supporting Information). An enhancement in prediction accuracy along with effective prevention of overfitting is observed when employing a noise‐aware model with the optimal noise level.

In order to show how the noise‐aware strategy (Strategy II) performs compared to existing active learning approaches that solely take the uncertainty caused by sparse data distribution into account, the efficiencies of the noise‐aware strategy (Strategy II) along with the noise‐unaware strategies with three acquisition functions from literature^[^ ^8^, ^30^, ^31^ ^]^ in searching for the ${\tilde{y}}_{i}$ with the minimum are evaluated and presented in Figure 2c–e. The formula of three acquisition functions are depicted in Table S3 (Supporting Information). The statistical results derived from 200 repetitions demonstrate that the noise‐aware strategy emerges as the most efficient one, requiring the minimal number of iterations (≈7) to attain the desired outcome. Noise‐unaware strategies require almost 4–5 times more iterations than the noise‐aware strategy to achieve the same outcome, as shown in Figure 2c. Furthermore, the noise‐aware strategy exhibits a marked decline in its opportunity cost curve of Figure 2d, thereby facilitating a swifter convergence toward the target. Figure 2e shows the probability density function (PDF) as the function of iteration count required to find the target in 200 trials. In every trial, Strategy II with SKO demonstrates a consistent ability to find the target material within 20 iterations. Conversely, strategy I with other acquisition functions typically require more than 20 iterations, with some instances necessitating over 50 iterations to achieve target discovery.

We also test the performances of both strategies on a ceramic database and a shape memory alloy database, showing that setting appropriate noise levels indeed improves the model's predictive capability. Furthermore, optimization tests on the two materials databases reveal that the noise‐aware strategy is more efficient than the noise‐unaware strategy, enabling the expedited discovery of materials with excellent performance with reduced experiments, particularly in scenarios where the model's predictive capabilities are limited, as detailed in Figures S6– S9 (Supporting Information). Given that, the noise‐aware strategy is further employed to develop SMAs with large latent heat.

### 2.2 Develop the High Temperature SMAs with Large Latent Heat via the Noise‐Aware Strategy

#### 2.2.1 Feature Selection and Virtual Space Construction with Guidance of Domain Knowledge

In this work, the major target property is large latent heat. As potential high‐temperature solid‐solid PCMs, the transformation temperature and the thermal hysteresis are paramount in practical applications. High transformation temperatures are essential for high‐temperature applications, while narrow thermal hysteresis is desired for precise control and sensitivity. Therefore, we consider domain knowledge and laboratory experience in feature selection and constructing the unknown materials virtual space to expect the optimization of multiple properties.

We gather a dataset comprising 65 SMAs, encompassing elements of Ti, Ni, Cu, Hf, Zr, Nb, Co, and Cr, all from our laboratory. To streamline the analysis, 23 features of physical and chemical properties describing the alloys are narrowed down by a filter from Pearson map and a wrapped method using Gradient Boosting. According to previous reports, the difference in atomic radii (δ*r*) among all components, Valence Electron Number (*VEN*) are the features closely related to the latent heat, thermal hysteresis and transformation temperature.^[^ ^8^, ^41^ ^]^ The finalized feature set includes Valence Electron Number (*VEN*), Ω parameter, Mixing Enthalpy (*H*), Difference of Atomic Radii (δ*r*), and the number of elements (*numa*). The details of database and feature selection are shown in Table S4, Figures S10 and S11 (Supporting Information). In the pursuit of novel alloys, we construct an unexplored virtual space relying on our experience knowledge from laboratory. As the Hf‐dopped alloy may lead to an excellent performance of high phase transformation temperature and relatively small thermal hysteresis,^[^ ^4^ ^]^ alloy system with formula of ${\mathrm{Ti}}_{100\%-u-x-y-z}{\mathrm{Ni}}_{u}{\mathrm{Hf}}_{x}{\mathrm{Cu}}_{y}{\mathrm{Cr}}_{z}$ is established. Here, the *u*, *x*, *y*, and *z* are allowed to vary in increments of 0.2%, adhering to constraints including 47.4% ⩽ u ⩽ 50.4%, 5% ⩽ x ⩽ 25%,0 ⩽ y ⩽ 5%,0 ⩽ z ⩽ 3%. This has yielded a vast set of 672 256 potential alloys.

#### 2.2.2 Estimation of Experimental Noise Level and Performances of Kriging Model

We utilize the method illustrated in Figure 1b to assess the noise level in our latent heat data. A set of noise level (τ^2^ = [ϕ(*y* ^*max*^ − *y* ^*min*^)]^2^) are assigned, where the maximum value *y* ^*max*^ is –9.86 J g^−1^, while the minimum value *y* ^*min*^ is –33.04 J g^−1^ in our dataset, and the prefactor ϕ ∈ (0, 1). The data noise‐aware Kriging models with different noise levels are built and the leave‐one‐out method is employed to calculate two error metrics of the model, allowing us to track the evolution of the model performance with the input of noise level τ^2^.

**Figure 3:**
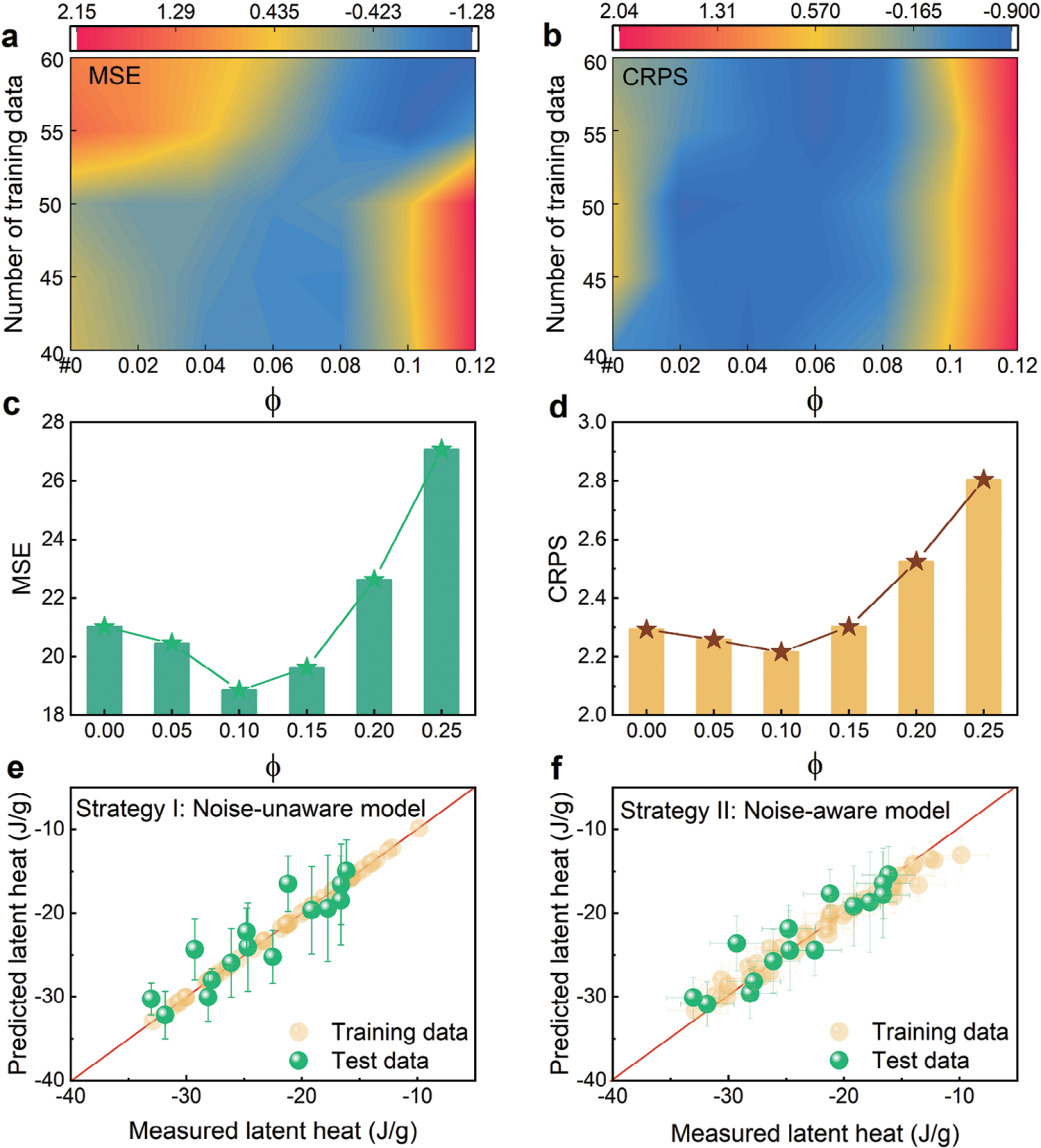
Performances of Kriging models with different preset noise level. a,b) Heat maps show standardized Mean Squared Error (MSE) and Continuous Ranked Probability Score (CRPS) as the function of preset noise level prefactor and data size. The darker color means a smaller error of the model. c,d) MSE and CRPS vary with different preset noise level prefactors. e,f) Performances of noise‐unaware model and noise‐aware model using leave out method.

We first investigate the two metrics change as a function of preset noise level prefactor ϕ and data size. The data size is controlled by randomly selected from the training set with a particular number. Such heat maps of standardized MSE and CRPS are shown in **Figure** **3**a,b, respectively. When ϕ is set to 0, implying no experimental noise consideration, the MSE and CRPS are relatively high. As the magnitude of noise level prefactor ϕ increases, both metrics exhibit a gradual decrease. However, if the noise level is set excessively high, the model experiences underfitting, resulting in a increase in MSE and CRPS. The tendency is quite similar for different size of data, that is, a best noise level exists for the model. Using all of our data, we found that a preset noise level prefactor of ϕ = 0.1 yields a minimum MSE of 18.86 and a minimum CRPS of 2.22, as respectively shown in Figure 3c,d. The noise‐unaware model yields a MSE of 21.03 and CRPS of 2.29. The noise‐aware model exhibits a percentage reduction of 10.32% in MSE compared to the noise‐unaware model.

The Kriging model inputting with the optimal noise standard deviation τ = 0.1 × (*y* ^*max*^ − *y* ^*min*^)= 2.318 J g^−1^ is constructed to predict the latent heat of the alloys in the virtual space using Equation (1) and provide predicted uncertainties through Equation (2). The performances of data noise‐unaware and data noise‐aware Kriging models are compared in Figure 3e,f. The improvement in the performance of data noise‐aware Kriging model is evident.

#### 2.2.3 Experimental Results Using Noise‐aware and Noise‐unaware Strategies

The estimated noise level in our experimental data allows for the implementation of the noise‐aware design loop depicted in Figure 1c to develop new SMAs with high latent heat. For comparison, the noise‐unaware design strategy in Figure 1c is also employed in parallel. Recommendations for specific compositions are made based on the predicted latent heat and the uncertainties associated with these predictions. Maximizing the utility scores ν_*SKO*_ (Equation 6) and ν_*EGO*_ (Equation 5) guides the selection for the noise‐aware and noise‐unaware strategies respectively. The selected composition is synthesized and measured, with the results fed back into the dataset to initiate a new iteration.

**Figure 4:**
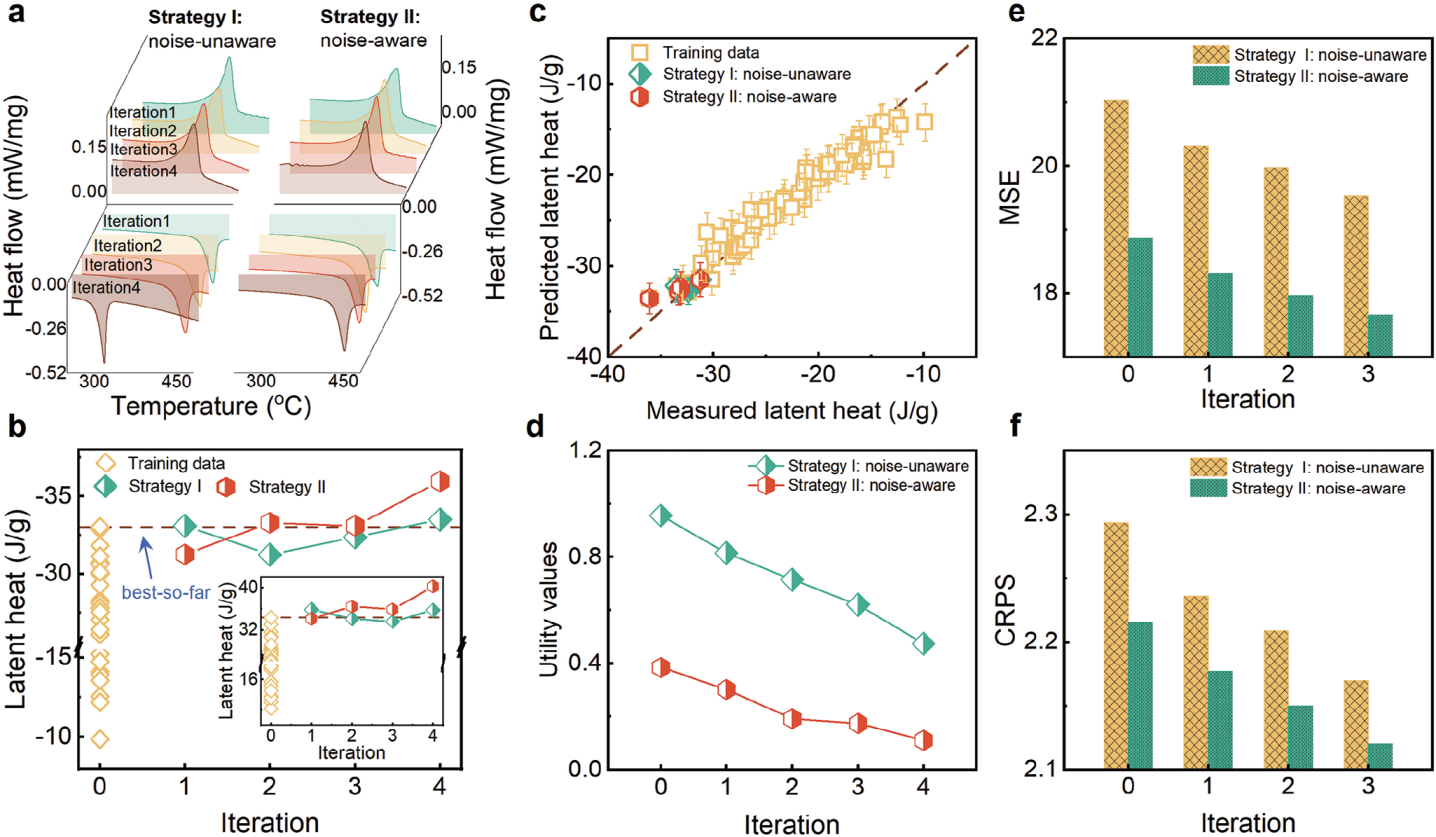
Experimental comparison between noise‐aware and noise‐unaware strategies. a) the DSC results of the alloys recommended by noise‐unaware strategy and noise‐aware strategy, b) latent heat of endothermic process and exothermic process, c) model performance based on original training data as well as newly added data, d) the maximum utility values vary with iterations, e,f) error metric MSE and CRPS of Kriging model after each iteration.

A total of four iterations are conducted for both the noise‐aware and noise‐unaware strategies. **Figure** **4**a shows the DSC curves obtained by differential scanning calorimetry for each new synthesized alloy, from which we obtain the phase transformation temperature and latent heat results determined by integrating the area under the endothermic peak, as detailed in Table S5 (Supporting Information). Figure 4b presents the endothermic latent heat and the exothermic latent heat (inset) as a function of the iteration number for both strategies. For each iteration, the noise‐aware strategy obtains higher latent heat values than the noise‐unaware one, except for the first iteration. From the second iteration onward, the recommendations from the noise‐aware strategy outperform the best‐so‐far results in the training data. After four iterations, the noise‐aware strategy led to the discovery of the Ti_0.266_Ni_0.484_Hf_0.25_ alloy with an endothermic latent heat of –36.08 J g^−1^, which is 3.04 J g^−1^ higher than the best value (–33.04 J g^−1^) in the original training dataset and 2.57 J g^−1^ higher than that of the Ti_0.322_Ni_0.488_Hf_0.19_ alloy(–33.51 J g^−1^) recommended by the noise‐aware strategy. The exothermic latent heat of this alloy reached 40.25 J g^−1^, surpassing the best reported value of 32.5 J g^−1^ for Ni_50.3_Ti_29.7_Hf_20_ alloy.^[^ ^4^ ^]^

We also monitor the performances of the noise‐aware and noise‐unaware models and the variation of maximum utility value as a function of iterations. The diagonal plot in Figure 4c reveals that the model predictions are basically consistent with the observation values and both strategies recommended alloys with high property values. As the number of iterations increases, the maximum utility score of ν_*SKO*_ and ν_*EGO*_ gradually decrease, as shown in Figure 4d. After four iterations, the maximum utility score of ν_*SKO*_ declines substantially to approximately zero, whereas the maximum utility score of ν_*EGO*_ remains 0.4740. This suggests that the noise‐aware strategy reaches the target more quickly. The MSE and CRPS as a function of iterations is plotted in Figure 4e,f respectively. The reduction in the MSE and CRPS with iterations indicates both models are gradually refined with the addition of new experimental results, even though only one data point is added at a time. The noise‐aware model consistently demonstrates lower error metrics for each iteration compared with the noise‐unaware model, highlighting a substantial enhancement in its prediction accuracy. Notably, the MSE of the refined noise‐unaware model at # = 3 decreases to 19.53, which is still higher than the MSE of 18.86 achieved by the noise‐aware model at its initial iteration (# = 0).

#### 2.2.4 Excellent Performances of TiNiHf Shape Memory Alloys

**Figure 5:**
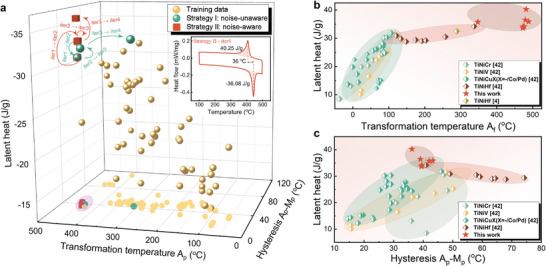
Figure of latent heat is plotted against transformation temperature and hysteresis. a) the distribution of the alloys within the functional space of latent heat, transformation temperature, and thermal hysteresis. b,c) Comparison of the properties of the newly developed alloys with those of previously reported alloys.

The properties of our newly developed alloys are measured and investigated. **Figure** **5**a illustrates the distribution of alloys developed in each iteration of the two strategies within the functional space of endothermic latent heat, transformation temperature, and thermal hysteresis. Thermal hysteresis is calculated as the temperature difference between the endothermic and exothermic peaks, while the transformation temperature is identified by the endothermic peak temperature (A_*p*_). Successive iterations show a clear trend toward higher latent heat for both strategies. Notably, all alloys exhibit transformation temperatures exceeding 438 °C and maintain thermal hysteresis within the range of 36−43 °C, except for the alloy recommended by the noise‐unaware strategy in the 4th iteration. These alloys stand out from the Pareto frontiers of these properties in the training dataset.

Figure 5b,c compare the properties of our newly developed alloys with those of previously reported solid‐solid phase change alloys.^[^ ^42^ ^]^ Whether considering the space of exothermic latent heat and austenite finish transformation temperature (Figure 5b) or the space of exothermic latent heat and thermal hysteresis defined by A_*p*_‐M_*p*_ (Figure 5c), the alloys developed from both strategies exhibit superior properties. They demonstrate larger latent heat, higher transformation temperatures, and lower thermal hysteresis compared to reported values. Notably, the alloy Ti_0.266_Ni_0.484_Hf_0.25_, discovered in the fourth iteration by the noise‐aware strategy, exhibits a thermal hysteresis of 36°C (A_*f*_‐M_*s*_ = 32°C), an endothermic latent heat of –36.08 J g^−1^ (exothermic latent heat of 40.25 J g^−1^), and a austenite finish transformation temperature (*A*_*f*_) of 481.7°C. The detailed DSC curve of alloy Ti_0.266_Ni_0.484_Hf_0.25_ and corresponding thermal properties are comprehensively presented in details in the inset of Figure 5a. In comparison to the best previously reported alloy, Ni_50.3_Ti_29.7_Hf_20_, which has an exothermic latent heat of 32.5 J g^−1^, a thermal hysteresis calculated by A_*f*_‐M_*s*_ of 31°C, and an *A*_*f*_ of 289°C,^[^ ^4^ ^]^ our newly developed alloy offers higher service temperatures and larger latent heat while maintaining a similar level of thermal hysteresis.

#### 2.2.5 Experimental and Key Feature Analysis of the Newly Developed Alloys

**Figure 6:**
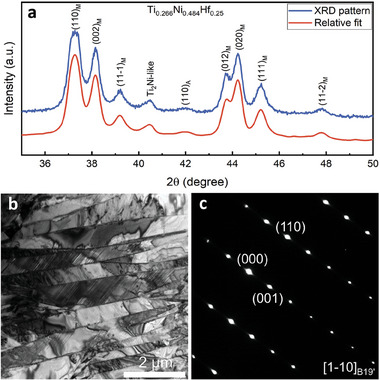
a) The XRD pattern of the Ti_0.266_Ni_0.484_Hf_0.25_ alloy. b,c) TEM observation and the selected area diffraction pattern (SAED) of B19' martensite in the Ti_0.266_Ni_0.484_Hf_0.25_ alloy.

To provide a fundamental understanding of the substantial latent heat observed in alloy Ti_0.266_Ni_0.484_Hf_0.25_, we perform qualitative analyses of the alloy's structural features utilizing X‐ray diffraction (XRD) and transmission electron microscopy (TEM) characterizations, as depicted in **Figure** **6**. Figure 6a shows the XRD pattern of the alloy Ti_0.266_Ni_0.484_Hf_0.25_ at room temperature, which can be well indexed according to the monoclinic B19' structure of martensite with lattice parameters a = 3.0868Å, b = 4.1003Å, c = 4.8756Å, β = 103.79°. A notable enlargement in the angle β indicates a significant difference in the geometry of the crystal lattice between the B19'martensite phase and B2 austenite phase. A large mismatch between the lattice structures of martensite and its parent phase may lead to high entropy change and, consequently, a substantial latent heat release during the transformation process.^[^ ^42^ ^]^ The TEM observation in Figure 6b and the corresponding selected area electron diffraction (SAED) in Figure 6c confirm the existence of B19' martensite. It is also evident that the monoclinic structure of the martensite differs significantly from the cubic structure of the parent phase according to the diffraction spots of B19' martensite, indicating a substantial energy difference during the phase transformation.

**Figure 7:**
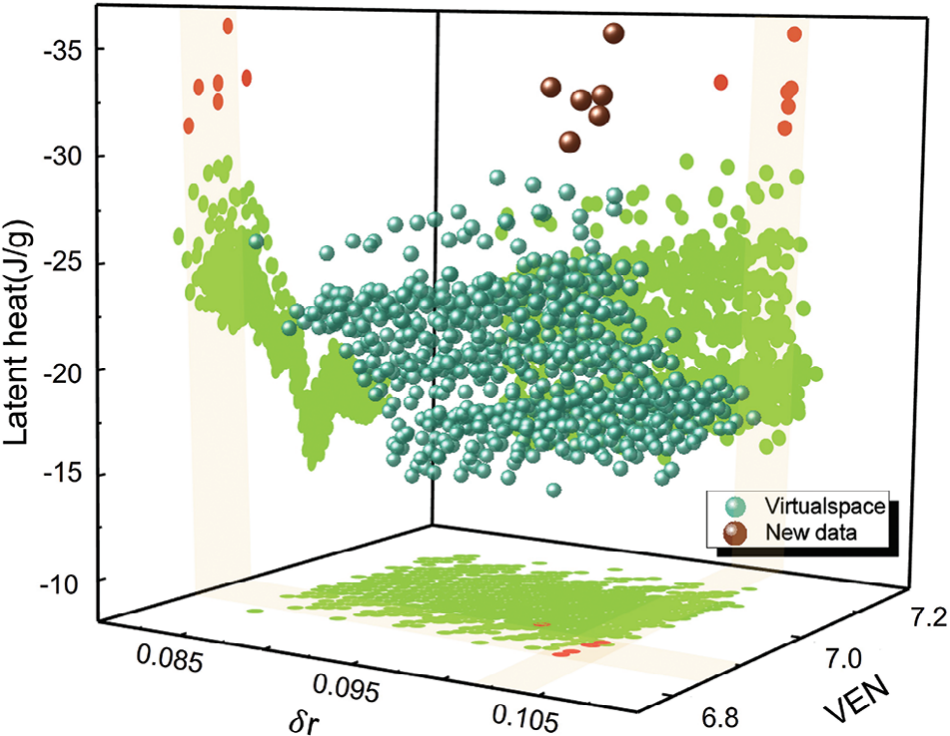
The measured latent heat during heating process varies with the materials features difference of atomic radii (δ*r*) and Valence Electron Number (VEN).

We also investigate the distribution of the newly developed alloys in the feature space, with the primary objective of identifying the specific features that have a pronounced impact on the performances of the alloys. **Figure** **7** visually depicts the distribution of the newly discovered alloys alongside a representation of the virtual space in terms of difference of atomic radii (δ*r*) and Valence Electron Number (*VEN*). The virtual space is represented by randomly sampling 2000 data points from the virtual space. The results indicate that the newly alloys exhibit large latent heat accompanied by relatively high values of δ*r* compared to those within the virtual space and the *VEN* values within the range of 6.8–7.0. Furthermore, as evidenced in Figure 5a, the newly alloys with aforementioned features also demonstrate relatively small thermal hysteresis, which aligns with previous research findings.^[^ ^8^ ^]^ This consistency with empirical domain knowledge suggests that the data‐driven strategy can effectively capture the underlying criteria for developing phase change alloys with large latent heat and small thermal hysteresis.

## 3 Conclusion

In summary, a noise‐aware active learning strategy is proposed, which combines noise level estimation, noise‐aware modeling, and noisy sample querying. By setting a series of noise levels, the one that yields the greatest enhancement in model performance is identified as the optimal noise level, which can approximate the true variance of the experimental data noise. With the incorporation of the optimal noise level, the noise‐aware Kriging model demonstrates higher accuracy compared to the noise‐unaware model. Followed by a noise‐augmented acquisition function to recommend the next alloy, the noise‐aware strategy demonstrates higher efficiency than noise‐unaware strategy in developing materials with the optimal properties. The feasibility and robustness of this strategy is validated in 1D and 3D mathematical functions as well as in two materials databases.

The noise‐aware strategy is applied to the development of shape memory alloys with large latent heat. It results in higher latent heat values for the newly synthesized alloys. Specifically, the alloy Ti_0.266_Ni_0.484_Hf_0.25_, discovered in the fourth iteration, exhibits an endothermic latent heat of –36.08 J g^−1^ during reverse martensite transformation, which is 9.2% higher than the best value (–33.04 J g^−1^) in the original training dataset. Additionally, benefiting from the incorporation of domain knowledge in feature selection and virtual space construction, this alloy selected has a high austenite finish temperature (481.71 °C) and relatively small hysteresis, making it suitable for LHTES applications in high‐temperature environments.

This work emphasizes the importance of noise level estimation and consideration in the modeling and querying process. If there is sufficient budget to support parallel tests, noise level can be estimated by calculating the variance of property values in repeated experiments. Alternatively, the noise level estimation approach in this work is particularly beneficial in material design scenarios where the target materials property is measured only once and no parallel results are available in the database.

## 4 Experimental Section

### Experimental Methods

The as‐cast ingots of the TiNi‐based alloys were flipped and melted six times by arc melting in argon atmosphere using a mixture of raw materials, pure Ti (99.99%), Ni(99.99%), and Hf(99.99%). Solution heat treatment was conducted in the argon atmosphere at 1000 °C for 1 h to ensure chemical homogeneity, followed by quenching in water at room temperature immediately. From each sample, the slices with the size of 3 mm × 3mm × 1 mm were cut using wire electrical discharge machining for differential scanning calorimetry (DSC) analysis. The temperature‐induced martensitic transformation behavior of the samples was measured using Netzsch 214 differential scanning calorimeter (DSC) with a heating/cooling rate of 10 °C min^−1^. Crystal structure of the sample was determined by a Bruker D8 ADVANCE X‐ray diffractometer (XRD) with Cu Kα radiation. The field‐emission transmission electron microscope (TEM, JEM‐F200) was employed to observe the morphology. The specimen for TEM observations was twin‐jet electro polished with a 10 vol.% HClO_4_ and 90 vol.% methanol solution at –15°C.

### Statistical Inference and Analysis

The data sets for the latent heat of SMAs are compiled from the results of the previous laboratory experiments. All experiments were performed under the same controlled conditions and protocols to minimize variability. The hartmann3 function was referenced within the DiceKriging package within the statistical studio *R*, and the Latin Hypercube Samling was accomplished utilizing the lhs package, also available within the *R* environment. The other two materials databases included in the Supporting Information, sourced from existing literatures, serve the purpose of validating the strategy. The Kriging model was utilized for estimating noise levels, making predictions, and evaluating the uncertainties of those predictions. This implementation was facilitated through the use of the DiceKriging package within the statistical studio *R* provided by Roustant et al.^[^ ^36^ ^]^

## Conflict of Interest

The authors declare no conflict of interest.

## Supporting information

Supporting Information

## Data Availability

The data that support the findings of this study are available in the https://github.com/tiany0321/noise-aware-strategy.
