# Supplementary material for: Noise‐Aware Active Learning to Develop High‐Temperature Shape Memory Alloys with Large Latent Heat
Source: Adv Sci (Weinh). 2024 Oct 3;11(44):2406216. doi: 10.1002/advs.202406216 (PMC11600200; doi:10.1002/advs.202406216)
Supplement: Supplementary file 1 — Supporting Information [file ADVS-11-2406216-s001.pdf]

ADVANCED SCIENCE Open Access

Supporting Information

for *Adv. Sci.*, DOI 10.1002/advs.202406216

Noise-Aware Active Learning to Develop High-Temperature Shape Memory Alloys with Large Latent Heat

Yuan Tian, Bin Hu, Pengfei Dang, Jianbo Pang, Yumei Zhou and Dezhen Xue**

Supporting information for Noise-aware active learning to develop high-temperature shape memory alloys with large latent heat

Yuan Tian*, Bin Hu, Pengfei Dang, Jianbo Pang, Yumei Zhou, and Dezhen Xue*

S1. ASSESSING THE ROBUSTNESS AND FEASIBILITY OF NOISE LEVEL ESTIMATION METHODS THROUGH MATHEMATICAL FUNCTIONS

To demonstrate the extent of proximity between the model prediction results and the ground truth following the incorporation of noise level into the Kriging model, a series of tests are conducted in a 1D mathematical function and 3D Hartmann3 function. The entire function space is discretized into 101 data points in 1D case and 400 data points in 3D case, and their true values y_i are obtained using **Equation S1** and **Equation S2**. The observed values $\tilde{y}_i = y_i + \varepsilon_i$ are constructed by introducing a random noise ε_i to the true values y_i, where ε_i of each candidate x_i is one realization of a noise random variable ϵ_i. We make the assumption that the noise random variable ϵ_i obeys a gaussian distribution $\mathcal{N}(0, \tau_i^2)$. If the noise level, denoted as τ_i^2, varies among the properties of different materials, it implies that the noise associated with each material's property adheres to unique Gaussian distributions, characterized by distinct variances. This type of noise, which exhibits varying statistical characteristics across different contexts, is referred to as heterogeneous noise. As the noise levels of the properties within the same materials system do not significantly diverge, we can reasonably adopt the notion of homogeneous noise to simplify our mathematical models and analysis procedures. By doing so, we can model the noise levels across all options as conforming to Gaussian distributions with a common variance. Here, we will discuss the prediction accuracy of the noise-aware model, examining its performance under scenarios where the true noise is either homogeneous or heterogeneous. If a homogeneous noise is supposed, τ_i is set as a constant with a value of $\tau_i = \phi_{real} \cdot (y^{max} - y^{min})$. Otherwise, the noise is heterogeneous with $\tau_i \sim \mathcal{N}(\phi_{real} \cdot (y^{max} - y^{min}), 0.07^2)$, where $\phi_{real} \in (0, 1)$ is a prefactor to control the magnitude of the true noise level. In this section, different values of ϕ are preset in $\phi \cdot (y^{max} - y^{min})$ to access the true noise level. $\phi \rightarrow \phi_{real}$ implies a proper noise level estimation.

1. *One-dimensional mathematical function*

To gain a clear visual understanding of how the noise level parameter affects prediction results, an investigation is undertaken using a case study involving a one-dimensional mathematical function. The expression of this function is shown as follows:

$$y = \frac{sin(8x)}{(1+x)} + 2cos(2x)x^3, x \in [0, 1] \tag{S1}$$

Homogeneous noise. The observed values $\tilde{y}_i = y_i + \varepsilon_i$ are obtained by sampling ε_i of each candidate x_i from the same Gaussian distribution $\mathcal{N}(0, (\phi_{real} \cdot (y^{max} - y^{min}))^2)$, where $\phi_{real} = 0.1$, $y^{max} = 0.8520$ and $y^{min} = -0.4754$. Five panels of **Figure S1** display the prediction results given by Kriging models with different preset noise level values $(\phi \cdot (y^{max} - y^{min}))^2$, where $\phi = 0.02, 0.1, 0.2, 0.25, 0.3$. As the noise level prefactor ϕ increases to 0.1, the prediction curve no longer accurately passes through the observed values. Instead, the distance between the prediction curve and the observed values with large deviations gradually increases, with the prediction curve approaching the true curve more closely. However, upon further increasing the prefactor ϕ to 0.3, the prediction performance starts to degrade again. In practical scenarios, modeling under the presence of noise interference confronts the problem of overfitting. By introducing a noise level parameter into the model, we can alleviate the issue of overfitting. As noise level increases, the model's performance undergoes from a state of overfitting to underfitting, with an optimal prediction state achieved at a critical value of the noise level prefactor $\phi_{opt} = 0.1$. When calculating the mean squared error (MSE) across all points, the noise-aware model with the noise level prefactor $\phi_{opt} = 0.1$ achieves the MSE of 0.0106 compared with the MSE of 0.1764 obtained by the noise-unaware model.

The capability to access various degrees of true noise levels is tested and the results are presented in **Figure S2**. Each time 15% data are randomly selected to train a Kriging model with a noise level, the mean squared error (MSE) and continuous ranked probability score (CRPS) are computed using all 101 data. The process is repeated 200 times, the mean values and 95% confidence intervals for the MSE and CRPS are shown in **Figure S2**(a) and (b). The true

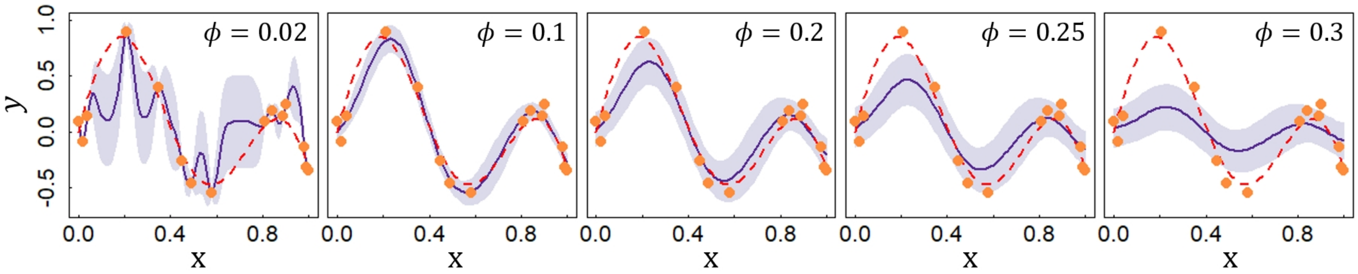

Figure S1 | Prediction outcomes of Kriging models incorporating different degrees of homogeneous noise level are presented. 15 solid yellow circles depict the noisy data utilized for training. The true function curve is shown in red dashed line, while the predicted means and their associated uncertainties are displayed in a purple line accompanied by a shadow.

noise level prefactors, denoted as ϕ_{real}, are assigned values of 0.05, 0.1, 0.15, and 0.2. As the true noise level in the data increases (noise level prefactor $\phi_{real} = 0.05 \rightarrow \phi_{real} = 0.2$), the model's predictive accuracy undergoes a significant decline. Utilizing Kriging modeling without noise level($\phi = 0$) will lead to a large MSE and CRPS, especially when the data suffers from a large true noise level $(\phi_{real} \cdot (y^{max} - y^{min}))^2$ with a prefactor $\phi_{real} = 0.2$. Modeling with the noise level reduces the MSE and CRPS obviously. The optimal noise level prefactors ϕ_{opt} that yield the minimum MSE and CRPS are pointed out by arrows. The optimal ϕ_{opt} closely approximating to the real noise level prefactor ϕ_{real} in most cases signifies a appropriate evaluation of the noise level. Except for the case $\phi_{real} = 0.2$, where there is a slight deviation in the estimated noise level, the estimation still remains close to the true noise level, and the model error obtained from the model using the optimal noise level is very similar to the model error achieved when the true noise level is used as input. Incorporating the optimal noise level into the model keeps the MSE at a relatively low level, indicating that estimating the noise level plays an important role in building a well-performing model, consistent with the content shown in **Figure S1**.

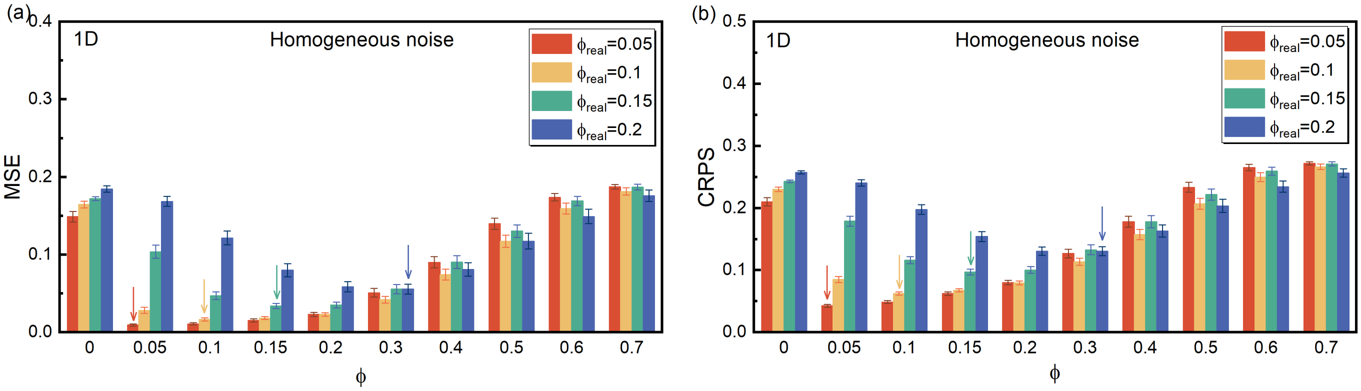

Figure S2 | (a) MSE and (b) CRPS as a function of the input noise level prefactor ϕ over 200 trials. Different colors represent different prefactors ϕ_{real} of the true noise level.

Heterogeneous noise. The observed values $\tilde{y}_i = y_i + \varepsilon_i$ are obtained by sampling ε_i of each candidate x_i from Gaussian distribution $\mathcal{N}(0, (\mathcal{N}(\phi_{real} \cdot (y^{max} - y^{min}), 0.07^2))^2)$, where $\phi_{real} = 0.1$. The outcomes predicted by Kriging models with different preset noise level values $(\phi \cdot (y^{max} - y^{min}))^2$, where $\phi = 0.02, 0.1, 0.15, 0.2, 0.25$, are shown in **Figure S3**. It can be seen that when the noise is heterogeneous, there are differences in the prediction results and their associated uncertainties compared to **Figure S1** with homogeneous noise. Among various predefined noise level prefactors ϕ, the prediction curve demonstrates the smallest deviation from the true curve when the value of ϕ is set to 0.1. Compared with the noise-unaware model exhibiting the MSE of 0.1831, the noise-aware model incorporating a noise level prefactor $\phi = 0.1$ demonstrates the MSE of 0.0178.

When heterogeneous noise is assigned to the true value, the statistical outcomes of MSE and CRPS over 200 trials are presented in the bar plots of **Figure S4**. As the noise level prefactor rises to a particular threshold, noticeable reductions in both MSE and CRPS are observed. Although the estimated noise level prefactor ϕ_{opt} does not always perfectly align with ϕ_{real}, it remains nearly close to it, resulting in MSE and CRPS values that are very proximate to the errors associated with ϕ_{real}. Estimation of noise levels and a comparative analysis of the performance between the noise-unaware model and the noise-aware model varying with different true noise levels in the case of the 1D function

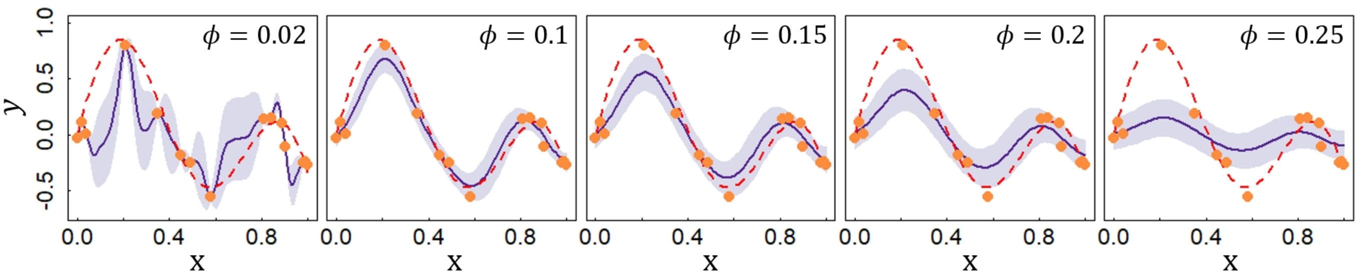

Figure S3 | Prediction outcomes of Kriging models incorporating different degrees of heterogeneous noise levels are presented. 15 solid yellow circles depict the noisy data utilized for training. The true function curve is shown in red dashed line, while the predicted means and their associated uncertainties are displayed in a purple line accompanied by a shadow.

is presented in **Table S1**.

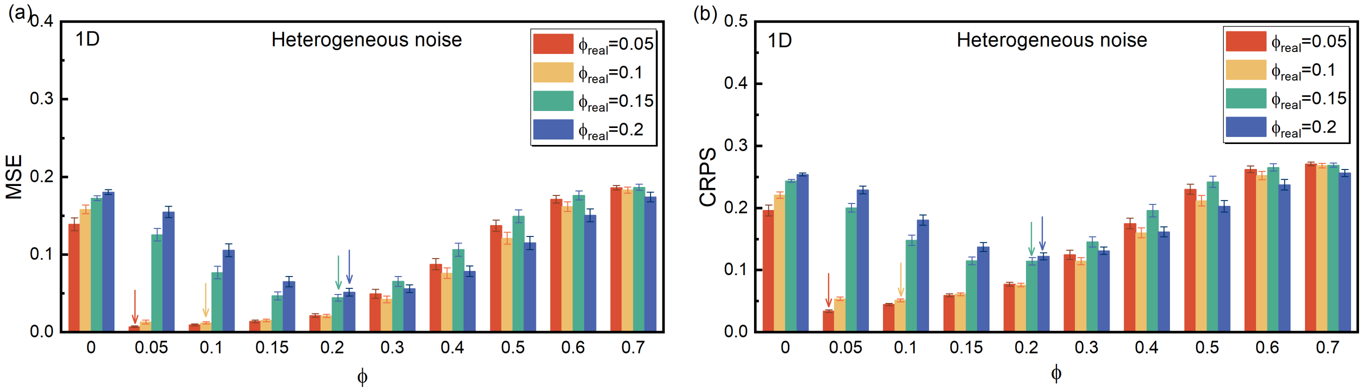

Figure S4 | (a) MSE and (b) CRPS as a function of the input noise level prefactor ϕ over 200 trials. Different colors represent different prefactors ϕ_{real} of the true noise level.

Table S1 | Estimation of noise levels and a comparative analysis of the performance between the noise-unaware model and the noise-aware model in the case of the 1D function.

Noise type	ϕ_{real}	ϕ_{opt}	SD_{opt}	$\text{MSE}_{\phi=0}$	$\text{MSE}_{\phi=\phi_{opt}}$	Difference in MSE	Percentage difference in MSE
Homogeneous	0.05	0.05	0.0664	0.1489	0.0094	0.1395	10.51%
	0.1	0.1	0.1327	0.1646	0.0165	0.1482	11.16%
	0.15	0.15	0.1991	0.1723	0.0340	0.1383	10.42%
	0.2	0.3	0.3982	0.1844	0.0556	0.1288	9.70%
Heterogeneous	0.05	0.05	0.0664	0.1391	0.0073	0.1318	9.93%
	0.1	0.1	0.1327	0.1583	0.0123	0.1461	11.01%
	0.15	0.2	0.2655	0.1728	0.0445	0.1283	9.67%
	0.2	0.2	0.2655	0.1806	0.0517	0.1289	9.71%

ϕ_{real} represents the true noise level prefactor; ϕ_{opt} represents the estimated noise level prefactor;
SD_{opt} represents the optimal noise standard deviation, which is calculated by $\phi_{opt} \cdot (y^{max} - y^{min})$;
$\text{MSE}_{\phi=0}$ represents the MSE of the noise-unaware model; $\text{MSE}_{\phi=\phi_{opt}}$ represents the MSE of the noise-aware model with the optimal noise level.
Difference in MSE represents the different of $\text{MSE}_{\phi=0}$ and $\text{MSE}_{\phi=\phi_{opt}}$.
Percentage difference in MSE is calculated by the formula: $\frac{MSE_{\phi=0} - MSE_{\phi=\phi_{opt}}}{MSE_{\phi=0}}$.

2. 3-dimensional hartmann3 function

We employ a well-known optimization test function, the Hartmann3 function, to generate data for a 3-Dimensional mathematical case characterized by the presence of multiple local minima and a global minimum. The function is defined by:

$$
\begin{aligned}
y &= -\sum_{n=1}^{4} \alpha_n exp(-\sum_{m=1}^{3} A_{nm}(x_m - P_{nm})^2), \\
where\ \alpha &= (1.0, 1.2, 3.0, 3.2)^\top \\
A &= \begin{pmatrix} 3.0 & 10 & 30 \\ 0.1 & 10 & 35 \\ 3.0 & 10 & 30 \\ 0.1 & 10 & 35 \end{pmatrix} \\
P &= 10^{-4} \begin{pmatrix} 3689 & 1170 & 2673 \\ 4699 & 4387 & 7470 \\ 1091 & 8732 & 5547 \\ 381 & 5743 & 8828 \end{pmatrix}
\end{aligned}
\tag{S2}
$$

The whole space is discretized into 400 points ($x_{mj}, m = 1, 2, 3; j = 1, 2...400$) using Latin Hypercube Sampling. Their corresponding y_j are evaluated by **Equation S2**. The maximum value y^{max} is -0.0007, while the minimum value y^{min} is -3.8353. From the entire dataset, 15% data are randomly chosen to train a Kriging model with noise level $(\phi \cdot (y^{max} - y^{min}))^2$, where the prefactor $\phi = 0, 0.05, 0.1, 0.15, 0.2, 0.3, 0.4, 0.5, 0.6, 0.7$. Subsequently, the MSE and CRPS are calculated using all 400 data points available. The statistical results of MSE and CRPS over 200 trails are represented in the bar plots of **Figure S5** when the heterogeneous noise is assigned to the true value. The true noise level can be nearly approximated, regardless of whether it is homogeneous or heterogeneous in nature. Estimation of noise levels and a comparative analysis of the performance between the noise-unaware model and the noise-aware model varying with different true noise levels in the case of the 3D function is presented in **Table S2**. In the 3-dimensional case, the percentage different in MSE is larger than in the 1D case. The accuracy of the noise-aware model is significantly improved when compared to the noise-unaware model.

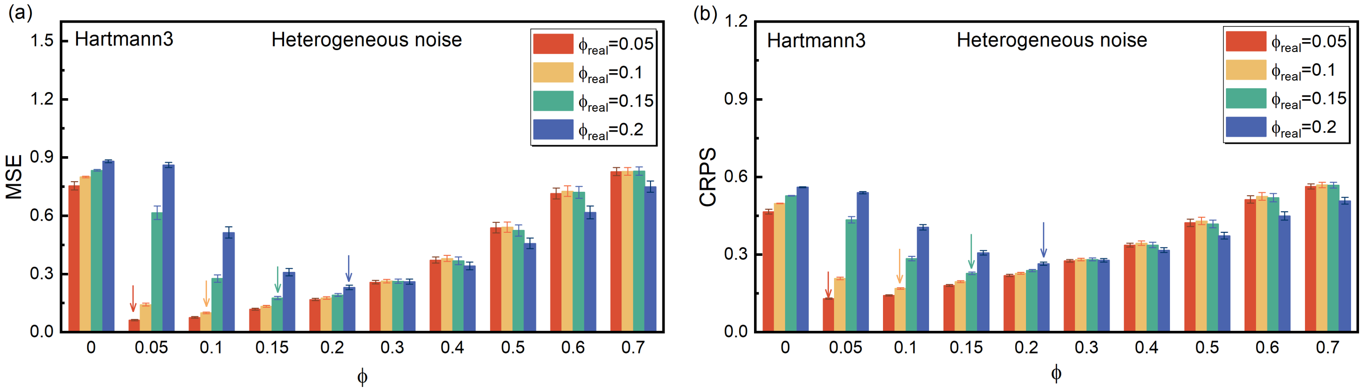

Figure S5 | (a) MSE and (b) CRPS as a function of the input noise variance prefactor ϕ over 200 trials. Different colors represent different prefactors ϕ_{real} of the true noise variance.

Table S2 | Estimation of noise levels and a comparative analysis of the performance between the noise-unaware model and the noise-aware model in the case of the 3D function.

Noise type	ϕ_{real}	ϕ_{opt}	SD_{opt}	$\text{MSE}_{\phi=0}$	$\text{MSE}_{\phi=\phi_{opt}}$	Difference in MSE	Percentage difference in MSE
Homogeneous	0.05	0.05	0.1917	0.7743	0.0592	0.7151	18.65%
	0.1	0.1	0.3835	0.8045	0.1097	0.6948	18.12%
	0.15	0.15	0.5752	0.8307	0.1706	0.6601	17.21%
	0.2	0.2	0.7669	0.8872	0.2686	0.6186	16.13%
Heterogeneous	0.05	0.05	0.1917	0.7549	0.0632	0.6918	18.04%
	0.1	0.1	0.3835	0.8006	0.1005	0.7000	18.26%
	0.15	0.15	0.5752	0.8350	0.1769	0.6581	17.16%
	0.2	0.2	0.7669	0.8818	0.2317	0.6501	16.95%

S2. NOISE-AWARE STRATEGY FEASIBILITY ASSESSMENT ACROSS DIVERSE MATERIALS DATABASES

A. Database of phase transformation temperature for shape memory alloys

Based on our preliminary research, we have compiled a database of reverse martensitic transformation temperatures for 130 shape memory alloys. The highest transformation temperature in the shape memory alloy (SMA) database is $y^{max} = 356.44^oC$, whereas the lowest temperature is $y^{min} = -102.78^oC$. For detailed information on the database construction, feature selection, and other specifics, please refer to the reference.[1] The optimal noise level is estimated to be $\tau^2 = [\phi_{opt}(y^{max} - y^{min})]^2$ through minimizing the model error shown in **Figure S6**(a) and (b), where the optimal prefactor ϕ_{opt} controlling the magnitude of the noise level is identified to be 0.02. 50% of these data are randomly selected as the training set, the transformation temperature of the remaining data are predicted using both the noise-unaware model and the noise-aware model. **Figure S6**(c) and (d) present the predictions along with the associated uncertainties provided by the two models, respectively. The noise-aware model with ϕ_{opt}=0.02 demonstrates significantly higher accuracy on the test data, with a significantly lower Continuous Ranked Probability Score ($CRPS_{test}$) of 16.26 and a notably higher coefficient of determination R^2_{test} of 0.91, when compared to the predictions obtained without considering noise level(Strategy I) with $CRPS_{test}$ of 24.10 and R^2_{test} of 0.83.

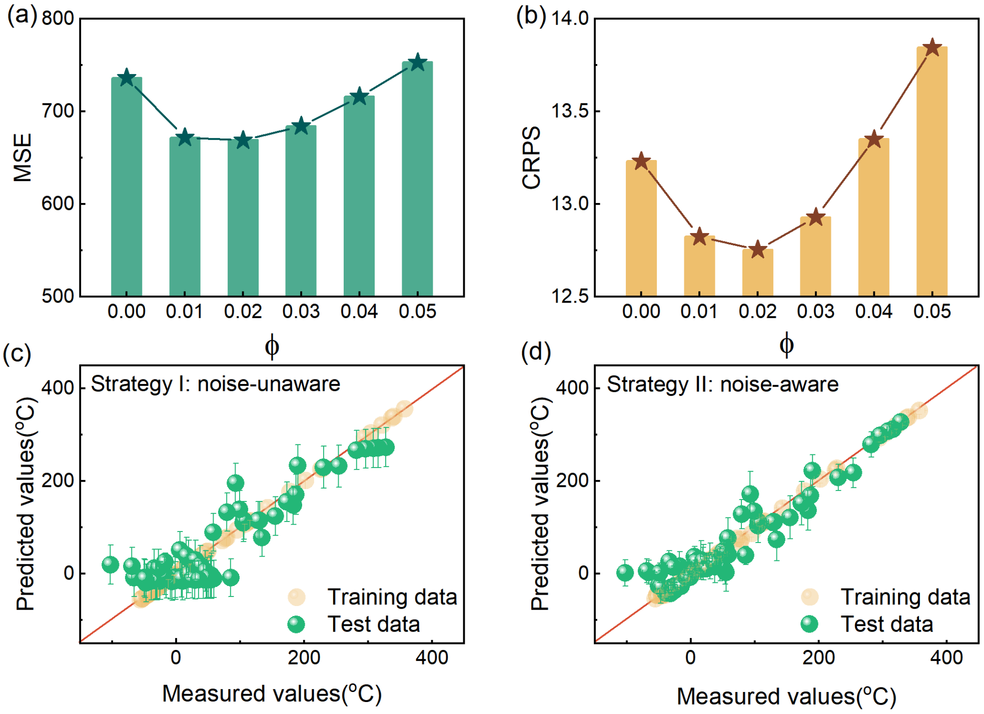

Figure S6 | Performance of Kriging models under various preset noise levels. (a-b) The Mean Squared Error (MSE) and Continuous Ranked Probability Score (CRPS) fluctuate with different preset prefactors of the noise levels. (c-d) Comparison of the performance between noise-unaware and noise-aware models when evaluated on test data.

Assuming that a subset of data, comprising $\gamma * n_{total}(\gamma = 0.4)$ elements, is randomly sampled from the entire database, the objective is to identify the alloy with the highest transformation temperature in the remaining data with the fewest iterations. The efficiencies of the Strategy I with different acquisition functions and Strategy II with the optimal noise level in searching for the alloy with the targeted temperature are evaluated. The three acquisition functions utilized in the noise-unaware strategy are Efficient Global optimization (EGO), Upper Confidence Bound (UCB) and Weighted ranking of predictions and uncertainties (WRPU), respectively. Their calculation principles and formulas are detailed in **Table S3**. The weights of R_μ and R_σ in acquisition function WRPU are assigned to $\alpha = 0.9$ and $\beta = 0.1$, respectively. The parameter λ of acquisition function Upper Confidence Bound (UCB) is set to 0.1. The statistical results derived from 200 repetitions are shown in **Figure S7**. Due to the high prediction accuracy of both the noise-aware model and the noise-unaware model, particularly for high-temperature data, the efficiencies of several strategies in identifying high-temperature alloys are relatively high. Each strategy requires only a few experiments on

Table S3 | Acquisition functions in noise-unaware active learning strategies

Acquisition function	Formula
Efficient Global Optimization (EGO)	$\nu_{EGO} = (\mu - y^{max})\Psi(\frac{\mu - y^{max}}{s}) + s\psi(\frac{\mu - y^{max}}{s})$ [2]
Upper Confidence Bound (UCB)	$\nu_{UCB} = \mu + 2log(\frac{DI^2\pi^2}{6\lambda}) \cdot s$ [3]
Weighted ranking of predictions and uncertainties (WRPU)	$\nu_{WRPU} = \alpha \cdot R_\mu + \beta \cdot R_s$ [4]

ψ is the probability density function of a standard normal distribution, Ψ is the associated cumulative distribution function. D is the total number of training samples. I denotes the current iteration number.
WRPU scores candidates by giving different weights on the rank of prediction mean(R_μ) and the associated uncertainty(R_σ). This acquisition allows for a balanced consideration of both prediction accuracy and prediction uncertainty in determining the overall ranking of candidates.

average to find the target SMAs. Notably, the noise-aware strategy emerges as the most efficient, necessitating the least number of iterations (~ 1.65) to attain the desired outcome. UCB is the least efficient strategy, requiring an average of 2.475 iterations to reach the target. Furthermore, compared with other methods, the noise-aware strategy exhibits a marked decline in its opportunity cost curve, thereby facilitating a swifter convergence towards the target. The opportunity cost (OC) curve of strategy II converges to 0 after an average of 3 iterations, strategy I with EGO requires 4 iterations, while strategy I with UCB and WRPU require an average of 7 iterations before converging. The Probability Density Function (PDF) distribution of strategy II is concentrated close to 0, indicating its high efficiency in most of the 200 trials. The long tails on the PDF curves of strategy I with different acquisition functions suggest that these methods may require more iterations to achieve the target in certain scenarios.

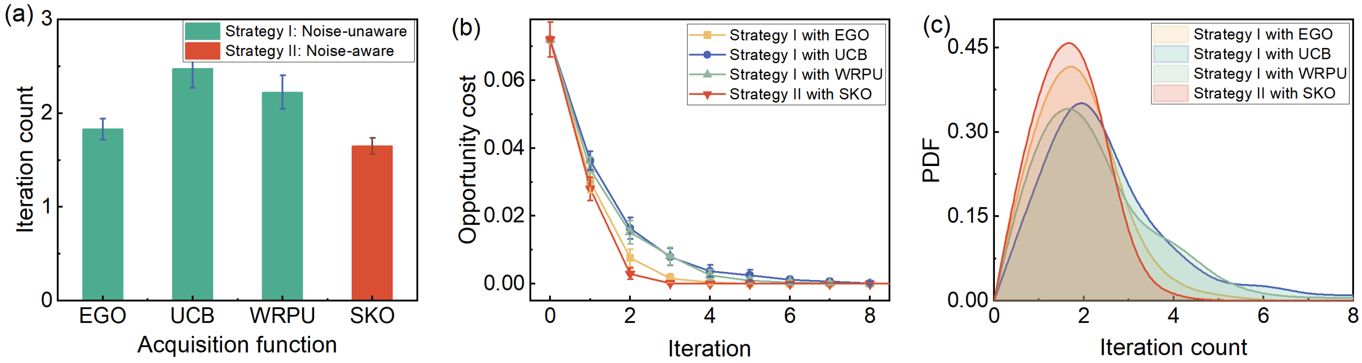

Figure S7 | The efficiencies of various active learning strategies in optimizing the transformation temperature of SMAs over 200 repeated trials. (a) The iteration count required to identify the alloy with the highest transformation temperature varies according to the method employed. The cyan bars depict the outcomes of noise-unaware strategies utilizing diverse acquisition functions, whereas the red bar represents the result achieved by the noise-aware strategy introduced in this work. (b) The opportunity cost changes as the iterations increase. A significant trend of declining opportunity cost signifies a faster approach towards the goal. (c) Probability density functions (PDF) are represented as functions of the iteration count. When the peak of the distribution is closer to an iteration count of 0, it indicates that the strategy demonstrates a higher level of efficiency in the majority of the 200 trials.

B. Database of piezoelectric coefficient for ceramic materials

The ceramic database consists of $n_{total} = 72$ samples, encompassing piezoelectric coefficient (d_{33}) along with the most crucial key features.[5] The highest d_{33} recorded within this database is $y^{max} = 610$ pC/N, while the lowest temperature is $y^{min} = 6$ pC/N. The noise estimation method proposed in this work is applied to this database, yielding the error bar charts depicted in **Figure S8**(a) and (b). The MSE of noise-unaware model (shown at $\phi = 0$) is large as 9844.85, while the noise-aware model shows its minimum MSE of 6952.18 as ϕ increases to 0.1. CRPS, which exhibits a similar trend to MSE, decreases from 57.07 to 47.33 when ϕ equals 0.1. Thus the noise level is decided as $\tau^2 = [0.1 \cdot (y^{max} - y^{min})]^2$. Furthermore, 40% of the data are randomly selected from the dataset as the training set. The remaining data serving as the test set are predicted using both the noise-aware model with the estimated noise level and the noise-unaware model. **Figure S8**(c) and (d) demonstrate that the performance of the noise-aware model on the test data is superior to that of the noise-unaware model. The test mean squared error (MSE_{test}), Continuous Ranked Probability Score (CRPS_{test}) and coefficient of determination (R^2_{test}) for the noise-unaware model are 7539.90, 52.98 and 0.50, while those for the noise-aware model are 6922.35, 47.62 and 0.55, respectively. In comparison to the case of shape memory alloy, the prediction model of the piezoelectric coefficient displays considerably lower prediction accuracy. Subsequently, we will study the efficiencies of active learning strategies in identifying the compound with the highest piezoelectric coefficient to show how these strategies perform in scenarios where the model's predictive accuracy is low.

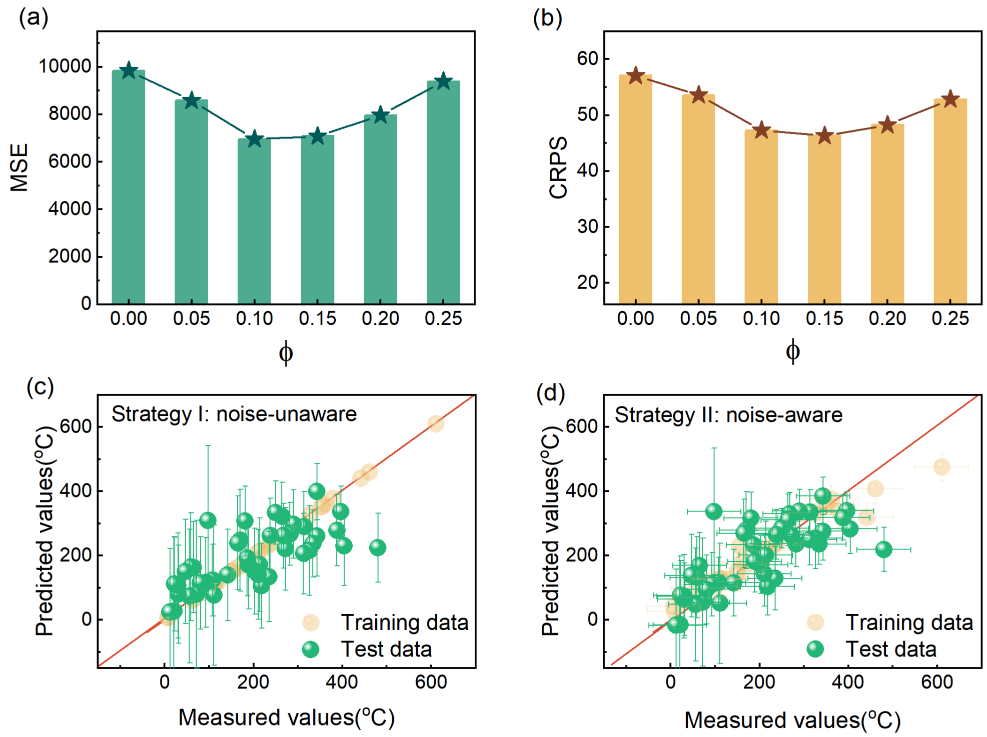

Figure S8 | Performance of Kriging models under various preset noise levels. (a-b) The Mean Squared Error (MSE) and Continuous Ranked Probability Score (CRPS) fluctuate with different preset prefactors of the noise levels. (c-d) Comparison of the performance between noise-unaware and noise-aware models when evaluated on test data.

A random selection of 40% data points serves as the training dataset, the target compound is welling to be sought among the remaining data using active learning strategies including noise-aware strategy and noise-unaware strategy mentioned above. The process is repeated 200 times, and the optimization efficiencies of four strategies are summarized in **Figure S9**. Similarly, among these 4 strategies, Strategy II with the optimal noise level prefactor $\phi_{opt} = 0.1$ exhibits the highest efficiency, requiring only 6.3 iterations to achieve the target. In contrast, Strategy I with WRPU needs 7.84 iterations, and Strategy II with UCB requires almost 13.9 iterations to reach the same objective. From the OC curve, it can be observed that Strategy II is able to approximate the target at a faster rate, whereas the piezoelectric coefficient of the compound recommended by Strategy I with UCB in the first 30 iterations is significantly far from the target, rendering it the least efficient. The fact that the PDF curve of Strategy II is closer to a distribution centered around 0 also indicates that this strategy is able to achieve the target within a small number of iterations in most cases

of the 200 trials. Noise-aware strategy demonstrates a relatively more pronounced advantage over other noise-unaware strategies compared to the shape memory alloy case. This indicates that noise-aware strategy is more capable of saving experimental costs, particularly when the model prediction accuracy is compromised. It is noteworthy that both UCB and WRPU are significantly impacted by parameters, resulting in relatively poor performance stability. When the parameter λ of UCB is adjusted from 0.1 to 200, the iteration count evaluated by noise-unaware strategy with UCB decreases from approximately 25 to 13.9. Therefore, it is proven that noise-aware strategy with SKO not only exhibits high efficiency but also good robustness in discovering high-performance materials.

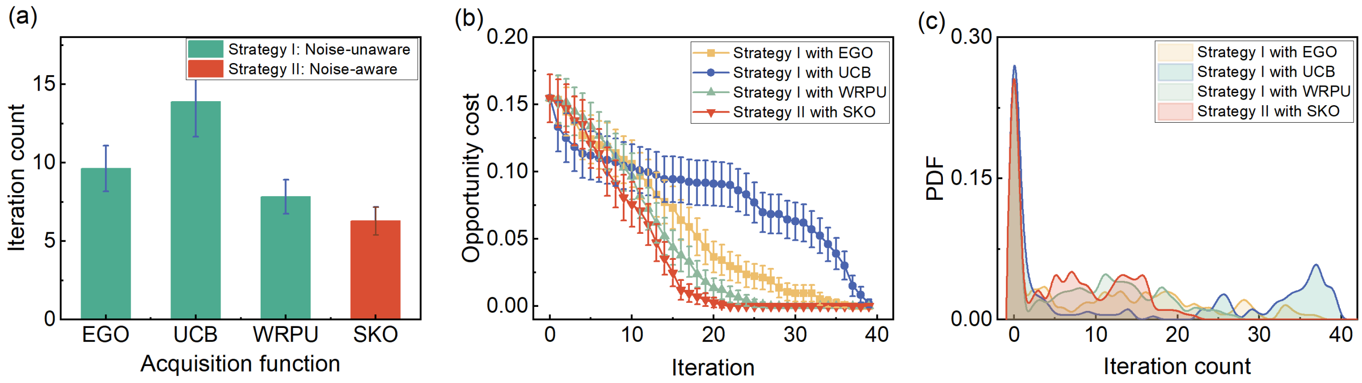

Figure S9 | The efficiencies of various active learning strategies in optimizing the d_{33} of ceramics over 200 repeated trials. (a) The iteration count required to identify the compound with the highest d_{33} varies according to the method employed. The cyan bars depict the outcomes of noise-unaware strategies utilizing diverse acquisition functions, whereas the red bar represents the result achieved by the noise-aware strategy introduced in this work. (b) The opportunity cost changes as the iterations increase. A significant trend of declining opportunity cost signifies a faster approach towards the goal. (c) Probability density functions (PDF) are represented as functions of the iteration count. When the peak of the distribution is closer to an iteration count of 0, it indicates that the strategy demonstrates a higher level of efficiency in the majority of the 200 trials.

S3. FEATURE SELECTION FOR LATENT HEAT MODELING

A total of 23 features, including those related to atomic information, thermodynamics, *etc.* have been constructed and are presented in **Table S4**. To prevent overfitting that arise from the irrelevant, noisy and redundant features, we employed two distinct feature selection methods to identify and select the most relevant features or material descriptors. Initially, Gradient Boosting (GB) is utilized to rank the 23 features according to their relative importance to the latent heat, and the top 15 features are chosen for further refinement in the subsequent filtering step (as depicted in **Figure S10**). A further down select is made by eliminating the features that display the high correlation among them, as identified through a Pearson correlation map. The Pearson correlation coefficient r for the above sets of features is calculated and visually presented as a color gradient heat-map shown in **Figure S11**. The red/blue indicates a negative/positive correlation between two features. The intensity of the color or the size of the pie indicates the strength of the correlation between the features, with a deeper color or larger size representing a stronger correlation. The features with Pearson correlation coefficient $r \geq 0.7$ are considered highly correlated and only one of them is retained. For instance, "*ven*" and "*cs*" are highly correlated and "*ven*" is chosen as the feature to be kept. Finally, 5 features Valence Electron Number (VEN), Ω parameter, Mixing enthalpy (H), difference of atomic radii(δr), the number of elements ($numa$) are chosen for latent heat prediction.

Table S4 | Material descriptors for Shape Memory Alloys

Material Descriptors	Physical properties	Formula
S	entropy of mixing of an n-element regular solution	$S = -R\sum_{i=1}^{n}(c_i lnc_i)$
H	enthalpy of mixing for multi-component alloy	$H = \sum_{i=1,i\neq j}^{n} 4H_{ij}c_ic_j$
Ω	predict the solid-solution formation	$\Omega = \frac{T_m S}{H}$
δr	difference in atom radii	$\delta r = \sqrt{\sum_{i=1}^{n} c_i(1-\frac{r_i}{r})^2}$
r	the atomic radius	$r = \sum_{i=1}^{n} c_i r_i$
x	electronegativity	$x = \sum_{i=1}^{n} c_i x_i$
δx	difference in electronegativity	$\delta x = \sqrt{\sum_{i=1}^{n} c_i(1-\frac{x_i}{x})^2}$
mismatch.x	mismatch of local electronegativity	$mismatch.x = \sum_{i=1}^{n}\sum_{j=1,i\neq j}^{n} c_ic_j * \lvert x_i - x_j\rvert$
Λ	Λ parameter	$\frac{S}{\delta r * \delta r}$
cs	Pettifor chemical scale	$cs = \sum_{i=1}^{n} c_i cs_i$
arc	Clementi's atomic radii	$arc = \sum_{i=1}^{n} c_i arc_i$
VEN	Valence Electron Number	$VEN = \sum_{i=1}^{n} c_i ven_i$
dor	Waber-Cromer's pseudopotential radii	$dor = \sum_{i=1}^{n} c_i dor_i$
numa	number of elements	$numa = n$
anum	atomic number	$anum = \sum_{i=1}^{n} c_i anum_i$
mass	atomic mass	$mass = \sum_{i=1}^{n} c_i mass_i$
volume	volume	$volume = \sum_{i=1}^{n} c_i volume_i$
Density	atomic density	$Density = \sum_{i=1}^{n} c_i Density_i$
Tm	melting point	$Tm = \sum_{i=1}^{n} c_i Tm_i$
YM	Young's Modulus	$YM = \sum_{i=1}^{n} c_i YM_i$
CE	Cohesive Energy (per atom)	$CE = \sum_{i=1}^{n} c_i CE_i$
EBE	Electron Binding Energy	$EBE = \sum_{i=1}^{n} c_i EBE_i$
ea	Valence Electron Number of average atomic number	$ea = \frac{VEN}{anum}$

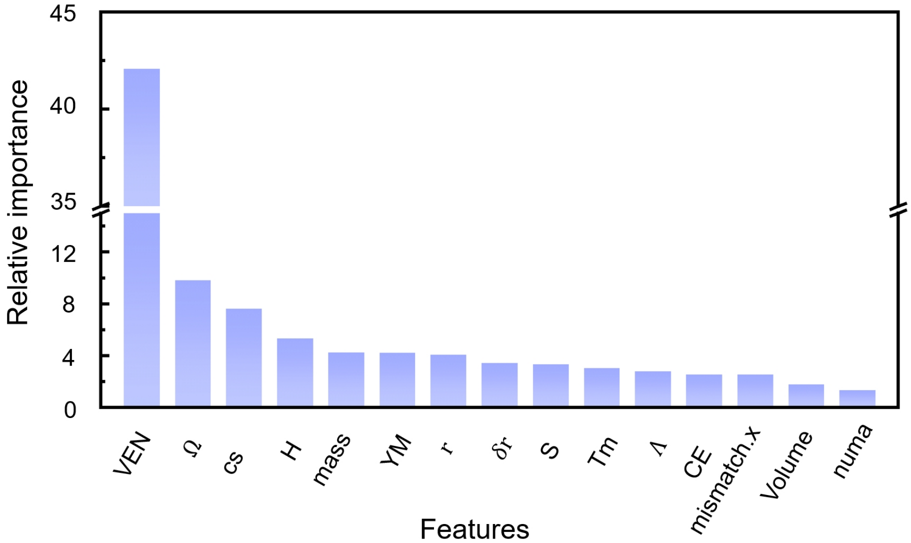

Figure S10 | The result of Gradient Boosting, which ranks the features according to their relative importance to the latent heat. Only the first 15 features are shown and selected.

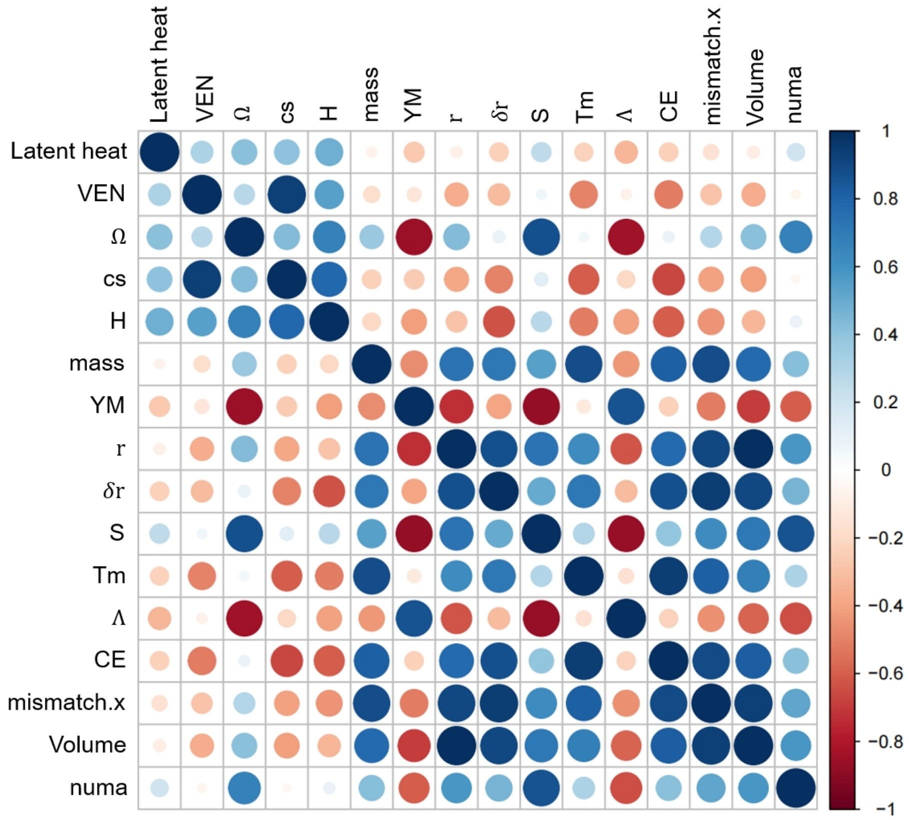

Figure S11 | The Pearson correlation map (pie chart), which further filters the highly correlated features (correlation coefficient $\geq$ 0.7).

Table S5 | Newly synthesized alloys recommended through two strategies and their thermal information.

Strategy	Iteration	Alloys	Q^{A-M}/J· g^{-1}	Q^{M-A}/J· g^{-1}	$\Delta T/^oC$	$T^{M-A}/^oC$	$T^{A-M}/^oC$
Noise-unaware	1	$Ti_{0.272}Ni_{0.478}Hf_{0.25}$	35.88	-33.11	43.37	451.47	408.1
	2	$Ti_{0.274}Ni_{0.476}Hf_{0.25}$	34.14	-31.25	40.35	447.29	406.94
	3	$Ti_{0.27}Ni_{0.482}Hf_{0.248}$	33.62	-32.37	39.7	438.53	398.83
	4	$Ti_{0.322}Ni_{0.488}Hf_{0.19}$	35.73	-33.51	42.05	309.38	267.33
Noise-aware	1	$Ti_{0.274}Ni_{0.476}Hf_{0.25}$	34.14	-31.25	40.35	447.29	406.94
	2	$Ti_{0.268}Ni_{0.482}Hf_{0.25}$	36.47	-33.3	39.31	443.79	404.48
	3	$Ti_{0.272}Ni_{0.478}Hf_{0.25}$	35.88	-33.11	43.37	451.47	408.1
	4	$Ti_{0.266}Ni_{0.484}Hf_{0.25}$	40.25	-36.08	36.1	442.18	406.08

Q^{A-M} represents the latent heat released during martensite transformation; Q^{M-A} represents the latent heat absorbed during reverse martensite transformation;
ΔT represents the hysteresis between martensite transformation and reverse martensite transformation, which is calculated by T^{M-A}-T^{A-M};
T^{M-A} represents the peak value of the transformation temperature during heating.
T^{A-M} represents the peak value of the transformation temperature during cooling.

References

[1] Y. Tian, R. Yuan, D. Xue, Y. Zhou, Y. Wang, X. Ding, J. Sun, T. Lookman, *Advanced Science* **2021**, *8*, 1 2003165.
[2] D. Xue, P. V. Balachandran, J. Hogden, J. Theiler, D. Xue, T. Lookman, *Nature Communications* **2016**, *7*, 11241.
[3] A. G. Kusne, H. Yu, C. Wu, H. Zhang, J. Hattrick-Simpers, B. DeCost, S. Sarker, C. Oses, C. Toher, S. Curtarolo, A. V. Davydov, R. Agarwal, L. A. Bendersky, M. Li, A. Mehta, I. Takeuchi, *Nature Communications* **2020**, *11*, 5966.
[4] Z. Rao, P.-Y. Tung, R. Xie, Y. Wei, H. Zhang, A. Ferrari, T. Klaver, F. Körmann, P. T. Sukumar, A. K. da Silva, Y. Chen, Z. Li, D. Ponge, J. Neugebauer, O. Gutfleisch, S. Bauer, D. Raabe, *Science* **2022**, *378*, 6615 78.
[5] R. Yuan, D. Xue, D. Xue, Y. Zhou, X. Ding, J. Sun, T. Lookman, *IEEE Transactions on Ultrasonics, Ferroelectrics, and Frequency Control* **2019**, *66*, 2 394.
